# Polymodal roles of transient receptor potential channels in the control of ocular function

**DOI:** 10.1186/s40662-015-0016-4

**Published:** 2015-03-02

**Authors:** Peter S Reinach, Weiwei Chen, Stefan Mergler

**Affiliations:** School of Ophthalmology and Optometry, Wenzhou Medical University, Wenzhou, Zhejiang 325027 P.R. China; Charité–Universitätsmedizin Berlin, Campus Virchow-Klinikum, Klinik für Augenheilkunde, Augustenburger Platz 1, D-13353 Berlin, Germany

**Keywords:** Transient receptor potential ion channels, Calcium, Retina, Cornea, Uvea, Lens

## Abstract

Maintenance of intracellular Ca^2+^ levels at orders of magnitude below those in the extracellular environment is a requisite for preserving cell viability. Membrane channels contribute to such control through modulating their time-dependent opening and closing behaviour. Such regulation requires Ca^2+^ to serve as a second messenger mediating receptor control of numerous life-sustaining responses. Transient receptor potential (TRP) channels signal transduce a wide variety of different sensory stimuli to induce responses modulating cellular function. These channels are non-selective cation channels with variable Ca^2+^ selectivity having extensive sequence homology. They constitute a superfamily made up of 28 different members that are subdivided into 7 different subfamilies based on differences in sequence homology. Some of these TRP channel isotypes are expressed in the eye and localized to both neuronal and non-neuronal cell membranes. Their activation generates intracellular Ca^2+^ transients and other downstream-linked signalling events that affect numerous responses required for visual function. As there is an association between changes in functional TRP expression in various ocular diseases, there are efforts underway to determine if these channels can be used as drug targets to reverse declines in ocular function. We review here our current knowledge about the expression, function and regulation of TRPs in different eye tissues in health and disease. Furthermore, some of the remaining hurdles are described to developing safe and efficacious TRP channel modulators for use in a clinical setting.

## Introduction

### TRP channel relevance to ocular function

One of the reasons why ocular transient receptor potential (TRP) channels are essential for maintaining normal vision is that some of their subtypes transduce environmental stresses into cell signalling events to control different physiological responses that alter cellular function. This realization has prompted intense research efforts to more extensively characterize how they control responses countering losses in cellular function caused by tissue injury, touch, fluctuations in ambient temperature, pH as well as osmolarity, hormonal exposure and pathogen tissue infiltration. Such insights can lead to the identification of lead compounds whose development may provide novel agents for improving the treatment of different ocular diseases in a clinical setting.

Functional TRP expression has been identified in every ocular tissue eliciting responses to a host of sensory stimuli under both physiological and pathophysiological conditions. By virtue of their non-selective cation permeability, their activation elicits intracellular Ca^2+^ transients ultimately triggering pain, scarring and inflammation to these various environmental activators [1,2]. There is heightening interest by drug companies in screening pipeline compounds targeting different ocular TRP channel subtypes because some of them are promising targets for reversing pathophysiological changes that compromise ocular function. For example, in different animal models of hypertensive glaucoma and injury-induced cornea scarring, declines in TRP function are associated with reductions in excitotoxic-induced retinal ganglion cell (RGC) dysfunction and corneal stromal fibrosis [3-5]. On the other hand, implying that suppressing TRP activation has therapeutic potential in glaucoma may be premature because of a concern about this model’s physiological relevance [1]. Another concern regarding the role of changes in functional TRP expression to visual processing stems from a more recent study showing that loss of TRP function instead inhibits optic nerve function [6]. These contradictory findings show that it is still unclear whether developing either novel TRP agonists or antagonists is the preferred approach for effectively treating glaucoma. This caveat prompts studies to unravel complexities of TRP channel involvement in ocular function. To shed some light on this complex question, we review literature describing ocular TRP channel functional expression in the cornea, conjunctival epithelium, uvea, lens and retina. In addition, the possible association between TRP channel mutations and some ocular diseases is considered.

## Review

### TRP channel nomenclature and channelopathies

Studies on Drosophila retinal phototransduction were the driving force for subsequent work on TRP channels. In the initial report, a mutant blind strain was identified in which a light flash elicited an aberrant transient rather than sustained retinal membrane depolarization [7]. Subsequently, it was realized that the channel eliciting this altered response has a homology with TRP channels. The TRP prototype was cDNA cloned from sensory neurons and functionally expressed nearly 30 years later [8]. This cloned channel is the forerunner of all subsequently identified TRP isoforms. Early on, it was referred to as the capsaicin receptor since this red pepper extract constituent is an agonist of this channel. This prototype has sequence homology with 28 different subtypes that together constitute a TRP superfamily [9,10]. In the TRP classification scheme, it is designated as TRP vanilloid 1 (TRPV1). All superfamily members assemble as tetramers and have a common configuration: six transmembrane spanning units in which the span between the fifth and sixth segments forms a conduit for cation membrane permeation. This superfamily is separated into seven different subfamilies and includes: a) TRPA (ankyrin); b) TRPC1-4 (canonical); c) TRPM 1–8 (melastatin); d) TRPML (mucolipin); e) TRPN (no mechano-potential); f) TRPP (polycystin); g) TRPV1-7 (vanilloid) [11]. Drosophila phototransduction is mediated by a TRPC channel together with a second TRPC channel, trp-like (TRPL) [12]. The relevance of defining TRP channel subtypes and their function is now more apparent since changes in their protein expression and function underlie either many hereditary diseases (so-called TRP *channelopathies*) or contribute indirectly to the genesis of several diseases including cancer [13-15].

### TRP activation overview

TRP channels are cellular sensors involved in the formation of sight, hearing, touch, smell, taste, temperature, and pain sensation. These diverse TRP functions are elicited through activation of downstream cell signalling pathways and allow the host cell to respond to benign or harmful environmental changes. The molecular mechanisms underlying TRP channel activation are very complex and not yet fully understood. TRP activation is caused by an increase in channel open time, which can be prolonged by a host of different factors. For example, changes in membrane stretch and exposure to an anisosmotic challenge (such as TRPV1 and TRPV4) as well as kinase-induced channel phosphorylation all lead to TRP activation whereas cooling directly can activate TRPM8 [16-18]. Another type of activation of TRPs occurs via more or less specific ligands. Initially, it was realized that either exogenous small organic synthetic compounds or natural products could activate TRPs. Even though TRPV1 was originally designated as the capsaicin receptor, subsequent studies revealed that it also interacts with other TRP isoforms. This red pepper extract constituent also activates voltage-gated inward and outward currents as well as impairs mitochondrial function and inhibits prostaglandin E2 production through non TRPV1 pathways [1]. There are indications in the retina that it may also activate TRPM1 channels. In addition, there may be other sites of interaction since in TRPM1^−/−^ mice; the responses to capsaicin were not fully attenuated. Other (synthetic) agonists are icilin (TRPM8), which was also described as a super cooling agent [19] or camphor (TRPV3) [20,21]. Furthermore, endogenous lipids or products of lipid metabolism are also ligands of TRPs. Finally, inorganic ions such as Ca^2+^ or Mg^2+^ can also (directly) activate TRPs (e.g. TRPM6 for Mg^2+^ [22] or TRPA1 for Ca^2+^ [23]). However, it is not yet known if the TRP conformational changes induced by Ca^2+^ or Mg^2+^ are the same as those induced by a thermal transition known to activate these different TRP channel subtypes.

Overall, classical activation of TRPs and in particular thermo-TRPs are mostly related to direct activation via mechanical stimuli or channel phosphorylation or certain exogenous and endogenous agents or inorganic ions. Given the complexities in identifying selective TRP channel modifiers, any drug target assignment should be verified by determining if a drug-induced response can be mimicked in transgenic mice having either a selective TRP gain or loss of function. If a drug effect cannot be replicated, it is possible that it will instead be caused by a compensatory response provided by modulation of another TRP subtype leaving drug selectivity uncertain.

### Reciprocal TRP channel and G-protein coupled receptor activation

G-protein-coupled receptors (GPCRs) and TRP channels are coexpressed on sensory neurons and act as sensors of noxious irritants and inflammatory stimulants. With approximately 850 GPCR variants in mammals, they are the largest family of signalling proteins. Due to their responsiveness to the largest group of diverse structural ligands, 30% of current drugs target GPCRs to treat pathophysiological conditions. More than 40 GPCR families contribute to pain processing and function as sensors on peripheral nerves to detect noxious, irritant, and inflammatory stimuli, including proteases, peptides, amines, and lipids [24]. Sensory nerves express TRP channels, which directly sense endogenous and exogenous chemical, mechanical, and thermal stimuli. These nonselective cation channels are also major downstream effectors of GPCR signalling. This GPCR-TRP axis is responsible for eliciting pain, itch, cough, and neurogenic inflammation [25-27]. Signalling pathways that emanate from the GPCR superfamily lead to altered TRP channel activity or expression. GPCR signalling can generate mediators that stimulate TRP channels (i.e., channel activation) or instead enhance their responses to TRP agonists (i.e., TRP sensitization). For example, TRPV1 activity can be modulated by a wide variety of endogenous small molecules through different regulatory mechanisms that either increase or decrease its sensitivity to different conditions known to either stimulate or inhibit this channel. In one case, TRP channels can amplify GPCR effects and mediate their contributions to transmission of pain, itch, cough, and neurogenic inflammation. Conversely, GPCR activation can generate mediators that stimulate TRP channels (i.e., channel activation) or that enhance their responses to TRP agonists (i.e., TRP sensitization). The net result is that TRP channels can amplify the effects of GPCRs and mediate their contributions to transmission of pain, itch, cough, and neurogenic inflammation [28].

### Dependence of TRP channel-induced phototransduction on GPCR activation

The mutant channel accounting for the aforementioned aberrant phototransduction response in the blind Drosophila strain was found in later studies to have a so-called TRP box structural sequence. This sequence is common to all TRPs including TRPV1, the founding member of the TRP superfamily. This 28-member superfamily forms the basis of receptor-operated TRP channel gating across species. In the plasma membrane of Drosophila photoreceptors, the receptor-operated channel activity requires GPCR activation and release of a heterotrimeric GTP bound G-protein complex, phospholipase C activity, and metabolism of phospholipids and phosphotidyl inositol-4,5 bisphosphate (PIP_2_) leading to the generation of inositol trisphosphate (IP_3_), membrane-associated diacylglycerol (DAG) and arachidonic acid. IP_3_ binds to its receptor on intracellular stores to release Ca^2+^ from intracellular stores (ICS) and DAG stimulates kinase activity. Ca^2+^ losses leading to the activation of store-operated Ca^2+^ channels (SOCs) are composed of TRP subunits such as TRPC1 and TRPC4 [29]. SOC activation results in the refilling of the depleted ICS. Although the mechanism is still not clear, the activation threshold for TRP channel activation is lowered (i.e. sensitized) by the influx of Ca^2+^ and fatty acid second messengers to permit channel opening leading to light-induced current and membrane depolarization. This process is desensitized by DAG-sensitive kinase phosphorylation of the TRP channel and the Ca^2+^ sensitive protein calmodulin (CaM), which binds to the channel preventing channel reactivation [30].

### Thermosensitive TRP channel activation

The ability to sense environmental temperatures and to avoid noxious heat or cold is crucial for the survival of all organisms. Six different thermosensitive TRP isotypes provide such protection in different ocular tissues. For TRPV1-4 [31] and TRPM8/TRPA1 [32-34] their activity is temperature sensitive within specific ranges. TRPV1-4 as well as TRPM8 and TRPA1 sensitivities span almost the entire temperature range to which mammals are exposed. In the vanilloid subfamily, TRPV4 and TRPV3 are activated by temperatures from 25 to 31°C, respectively, whereas TRPV1 is activated at 43°C and TRPV2 at a noxious temperature of 51°C [35]. On the other hand, TRPM8 and TRPA1 are activated below 25 and 17°C, respectively. The mechanism(s) underlying these different temperature sensitivities are poorly understood.

Three hypothetical mechanisms may account for the origin of these remarkably steep temperature sensitivities. They include: 1) steep specific temperature dependence for ligand binding; 2) temperature-induced channel rearrangement; 3) temperature-dependent membrane tension changes may lead to channel opening [31]. A recent report suggests that differences in TRP channel protein sequence endow thermal sensitivity, which could mean that this property is ascribable to specific structural changes. This suggestion stems from the finding that in the Drosophila TRPA1 isoform, thermal sensitivity variability is linked to changes in the expression of a specific 37-amino-acid long intracellular region (encoded by a single exon) [36]. TRPM8 stimulation could result in graded shifts of voltage-dependent activation to more negative membrane voltages [37]. This channel may be also regulated by GPCRs. One indication for such an interaction is that both activation of a GPCR and a nerve growth factor receptor inhibited menthol- and cold-induced TRPM8 activity [38]. Using temperature sensitivity as a criterion for channel identification may be more reliable than relying on drug sensitivity due to uncertainties of drug selectivity. Another reason for assessing changes in thermosensitive TRP channel expression is that they may underlie sensitivity differences in sensory neurons to cold and mechanical stretch [39]. Such a possibility has prompted interest in developing drugs targeting thermosensitive TRP expression since they may prove to be useful antinociceptives.

### TRPs in ocular tissues and cells

#### Retina, pigment epithelium and uvea

Retinal TRPV1 gene transcripts and protein expression have been identified in species ranging from teleosts, amphibians, birds to mammals. Even though capsaicin is routinely used to characterize functional TRPV1 expression, there is uncertainty about its selectivity since it has side effects. Capsaicin may also activate a TRP melastatin receptor (i.e. TRPM1) described in the retina [40]. However, this is questionable since capsaicin also elicited current responses in TRPM1^−/−^ mice [41]. A recent report showed that the capsaicin response is eliminated in mice lacking the mGluR6 receptor. This suggests that this agonist may also act on either this metabotropic receptor or its downstream GTP binding protein (G_0_) heteromers [42]. The caveat of sole reliance on capsaicin effects to identify TRPV1 activity also applies to capsazepine (CPZ), which is commonly used as a TRPV1 antagonist. This cautionary note is indicated since CPZ also has effects in TRPV1^−/−^ mice. These responses include antagonism of voltage-gated Ca^2+^ channels, acetylcholine receptors, hyperpolarization-activated cation channels, and stimulation of amiloride-sensitive ENaCs [1]. Another cautionary note is indicated regarding protein expression since with different antibodies and protocols, the number of reported bands is inconsistent. This variability points to the need to validate all results by showing negative staining in Western blots in fractions obtained from TRPV1^−/−^ mice. Nevertheless, TRPV1 expression may be involved in controlling ontogenetic development. It may regulate the synaptic plasticity during superior colliculus development [1]. Despite these uncertainties, maximal activation of TRPV1 by capsaicin has been shown to induce neuronal and glial excitotoxicity in some areas of the brain and apoptosis in cultured RGCs. Based on some studies suggesting that TRPV1 is mechanosensitive, Sappington et al. determined if TRPV1 is a target for RGC death *in vivo* during exposure to elevated intraocular pressure (IOP). As raising the pressure in an isolation chamber by 70 mmHg caused increased RGC death, the authors surmised that elevated intracellular Ca^2+^ levels were attributable to neurotoxicity caused by TRPV1 hyperactivation [3]. These data provide an interesting retinal disease model for RGC remodelling and apoptosis elicited by increases in intracellular Ca^2+^ levels during exposure to elevated pressures that induce mechanosensitive TRPV1 hyperactivation.

Even though TRPV1 hyperactivation may be deleterious under some conditions, there are contradicting reports showing that this change is instead neuroprotective in other contexts. Specifically, in TRPV1^−/−^ mice, elevated IOP was more damaging to RGCs when compared to the wildtype counterparts, suggesting that TRPV1 expression is instead neuroprotective [6]. Furthermore, in CPZ-treated rats, anterograde retrograde transport of markers were inhibited, which was associated with axonal loss and astrogliosis. These findings prompted suggestions that TRPV1 expression is instead neuroprotective during RGC exposure to retinal stress. Taken together, these opposing effects of alleged changes in TRPV1 activity may be context dependent.

Various other TRPs are also detected in the mouse retina (TRPC1-4 TRPM1/3/7, TRPML1, TRPP2, TRPV2, TRPV4 [43,44]. In mouse RGCs, TRPV4 modulates calcium flux, spiking rate, and apoptosis. Furthermore, TRPC1 and TRPC4 are expressed in the chicken retina [45]. TRPM1 is expressed on ON-bipolar cell dendrites that invaginate photoreceptor terminals and on the synaptic ribbons of a subclass of rods. This expression pattern suggests a dual function for TRPM1 in the ON-pathway [40]. TRPV1, TRPM8 and TRPA1 are expressed in retinal tumour cells (retinoblastoma). Interestingly, the expression of TRPA1 was completely suppressed in a retinoblastoma cell line, which is resistant to the cytostatic agent etoposide [46]. In addition, TRPV1-4, TRPM8 and TRPA1 expressions in retinal pigment epithelial (RPE) cells are described in a review describing ocular tissue specific gene expression patterns [47]. Increasing the ambient temperature or insulin-like growth factor-1 (IGF-1) increased the vascular endothelial growth factor-A (VEGF-A) secretion rate in RPE cells indicating that TRPs are involved in the IGF-1 induced response [48]. Regarding the human uvea, there is only one study demonstrating gene expressions for TRPV1, TRPM8 and TRPA1. Notably, in human uveal melanoma cells, the gene expression of TRPM8 is at a lower level whereas the TRPA1 expression is at a high level in healthy uvea [49].

#### Conjuncitva and limbus

There are functional thermosensitive TRPM8, TRPV1 and TRPV2 as well as TRPV4 expressions in conjunctival epithelial cells [50,51]. Functional TRPM8 expression is evident based on increases in intracellular Ca^2+^ levels and currents elicited by icilin that were blocked by the TRPM8 antagonist, BCTC. Similarly, BCTC also blocked these increases induced by a thyroxin metabolite, thyronamine (T1AM), indicating that it is an endogenous TRPM8 agonist. TRPM8 activation by either icilin or T1AM suppressed TRPV1-induced increases in interleukin 6 (IL-6) expression indicating intracellular signalling interaction or crosstalk between these TRP channels. TRPV1 expression is functional since capsaicin-induced Ca^2+^ transients, ionic currents and increases in proinflammatory IL-6 release were all blocked during exposure to CPZ. TRPM8 and TRPV1 coexpression in these cells as well as on afferent trigeminal ophthalmic branch sensory neurons is supportive of the notion that both of these receptors contribute to the maintenance of ocular surface health [52-55]. Cold stimulation of TRPM8 increases in mice lacrimation whereas TRPV1 activation by noxious agents induces pain and avoidance behaviour [56,57]. On the other hand, the TRPV1-induced increase in proinflammatory cytokine release occurring in dry eye disease (DED) may be moderated through receptor crosstalk between TRPM8 and TRPV1 [50]. Therefore, it may be advantageous to evaluate the effectiveness of TRPM8 agonists to increase lacrimation and reduce TRPV1-induced immune responses.

#### Corneal epithelium

In rats, mice and human corneal epithelia, there are TRPV1-4 expressions [58-60]. Their presence suggests that this tissue layer is well poised to counter challenges to its functional competence caused by exposure to noxious agents, anisosmotic challenges, elevated temperatures and pathogens. Furthermore, the mitogenic response to epidermal growth factor receptor (EGFR) phosphorylation by EGF is dependent on increases in plasma membrane Ca^2+^ influx elicited by a SOC composed of TRPC4 subunits. This dependence is evident since knockdown of TRPC4 expression in human corneal epithelial cells (HCEC) markedly reduced cell proliferation and migration [61]. Conversely, another important TRPV1 function is that its activation by capsaicin induces increases in cell proliferation and migration through transactivating EGFR [62]. The HCEC mitogenic response to capsaicin is consistent with a mouse study in which corneal re-epithelialization following debridement was delayed in TRPV1^−/−^ knockout mice compared to their wildtype counterparts. This stimulatory effect of TRPV1 in promoting cell proliferation and migration is associated with increases in IL-6 and substance P expression, which are coactivators of growth factor induced wound healing [58]. TRPV4 expression was identified in intact human corneal epithelium and its activation by exposure to a hypotonic challenge is required for inducing regulatory volume decrease behaviour [63]. Furthermore, hypertonic stresses identical to those described in some dry eye patient tears elicited through TRPV1 channel activation, raised proinflammatory cytokine levels by eliciting mitogen-activated protein kinase (MAPK) and nuclear factor-κB (NF-kB) activation [64,65]. Taken together, corneal epithelial functional TRP expression contributes to countering anisosmotic challenges similar to those identified in the tears of some dry eye patients. Such stress can compromise maintenance of corneal epithelial barrier function rendering the tissue layers vulnerable to pathogenic infiltration and declines in transparency.

#### Corneal stroma

Subsequent to the breaching of the corneal epithelial basement membrane by a severe injury, transforming growth factor–β (TGFβ) infiltrates into the stroma and activates its cognate receptor on human fibroblasts also expressing TRPV1. These two receptors interact through crosstalk to induce myofibroblast transdifferentiation leading to fibrosis and corneal opacification subsequent to a severe injury [66]. This dependence on TRPV1 activation is evident since during exposure to CPZ, exogenous TGFβ-1 failed to elicit increases in α-smooth muscle actin (SMA) expression. This result is pertinent for efforts to reduce corneal opacification and inflammation following a chemical burn since it suggests that in a clinical setting treatment, a TRPV1 antagonist could promote restoration of corneal transparency by inhibiting TGFβ-induced myofibroblast transdifferentiation. It is noteworthy that loss of TRPV1 function in two different mouse wound healing models has opposite effects on restoring transparency during corneal wound healing. In the epithelial layer, wound closure is delayed due to declines in cell proliferation and migration whereas in the stroma, fibrosis and inflammation are suppressed. These polymodal effects of TRPV1 activation suggest that TRPV1 antagonist usage in a clinical setting may need to be restricted to cases involving penetrating stromal injury in which case corneal opacification occurs due to fibrosis and dysregulated chronic inflammation [67].

#### Corneal nerve fibres

TRPA1 and TRPM8 are expressed on corneal afferent nerves. Corneal nerve fibres are radially distributed within the stroma and the adjacent limbus [68]. Mouse, guinea pig and human corneal nerve fibres contain sensory neurons in which TRPV1 and TRPM8 are highly expressed at levels similar to those in their non-corneal counterparts [54,55,57] and in non-corneal primary sensory neurons [35]. These channels mediate excitation and subsequent desensitization to capsaicin (TRPV1) or menthol (TRPM8). In a clinical context, thermosensitive TRPM8 function in nerve fibres may be relevant for dry eye syndrome treatment since corneal cooling below 23°C increased lacrimation through TRPM8 activation whereas warming had an opposite effect [57].

#### Corneal endothelium

Rae and Watsky [69] first described that in the corneal endothelium, whole-cell configuration of patch-clamp recordings showed an outwardly rectifying thermosensitive current along with a K^+^-selective current. The origin of this thermosensitive component was not identified but may be attributable to TRP activity [69]. More recently, thermosensitve TRPV1-4 channel activities were identified in human corneal endothelial cells [70-72]. However, the functional importance of their presence is still unclear. Thermosensitive TRPM8 was identified in human corneal endothelial cells based on showing that application of the super cooling TRPM8 agonist icilin reversibly increased intracellular Ca^2+^. One possible upshot of TRPM8 presence in the endothelium is that preservation of its function in an eye bank setting may be dependent on its activation during storage at temperatures below 23°C [73,74]. Such expression may contribute to the ability of the endothelial cell layer to maintain tissue homeostasis function over a broader temperature range. Besides TRPM8 expression in human corneal endothelial cells, there might also be other TRPs expressed in human corneal endothelium since H_2_O_2_ induced significant increase in [Ca^2+^] [75]. These limited results indicate that there remains a large void in our understanding of the contribution made by functional TRP expression to the maintenance of corneal transparency and deturgescence.

#### Lens epithelium

Regarding lens (epithelial) cells, there is very limited information on the functional involvement of TRPs. TRPV4 in porcine lens epithelium regulates hemi-channel-mediated ATP release and Na^+^-K^+^-ATPase activity [76]. In addition, TRPV1 gene expression was identified in the lens and its gene expression is at a very high level compared to that in the retina [77].

### TRP channelopathies and ocular disease

TRP gene mutations underlie numerous inherited diseases in humans including the eye. These diseases are associated with dysfunctional channel expression and are termed *TRP channelopathies* [13,14,78]. So far, TRP channelopathies have been linked to cardiovascular, renal, skeletal and nervous system pathologies [13]. With respect to the eye, there are only a few such studies. One of them deals with mucolipidosis type IV (MLIV). It is an autosomal recessive, neurodegenerative lysosomal storage disorder, which is due to mutations in the gene MCOLN1. MLIV is clinically characterized not only by ophthalmologic abnormalities such as corneal opacity, retinal degeneration and strabismus, but also by other non-ocular abnormalities. MCOLN1 which encodes the protein mucolipin 1 (MNL1) is a non-specific cation channel (TRPML1) [79]. In addition, human TRPM1 mutations are associated with congenital stationary night blindness (CSNB), whose patients lack rod function and suffer from night blindness starting in early childhood [80].

#### Dry eye syndrome

At this point, it is unclear whether there is an association between a TRP channelopathy and DED. Nevertheless, even without a mutated expression, an altered level of expression may trigger or promote DED. Based on current evidence, there are indications that either one or both of the aforementioned changes could contribute to DED. In DED, ocular surface inflammation and desiccation may stem from dysfunctional TRPV1 and TRPM8 expressions and/or conditions in this disease that promote their hyperactivations. With regards to TRPV1, it is activated by hypertonic conditions similar to those identified in some DED afflicted patients. *In vitro*, such levels lead to increases in the expression of the same proinflammatory cytokines, chemokines and matrix metalloproteinases, whose levels are elevated in patient tears. TRPM8 is also implicated since its expression on afferent sensory corneal nerves and in the cell layers contributes to maintenance of basal tear flow during thermal stress [51,57,64]. Taken together, TRPM8 agonists should be evaluated in various dry eye animal models to determine if they reduce dry eye symptomology and dampen TRPV1-induced inflammation and corneal opacification.

#### Diabetic retinopathy

In diabetic retinopathy, impaired blood flow due to vascular occlusion can lead to retinal hypoxia causing photoreceptor and neuronal cell death and visual impairment. There are various studies describing a connection between TRPs and diabetes mellitus [81,82], but none directly relates to diabetic retinopathy. On the other hand, some are available pertaining to non-diabetic related retinopathy (e.g. melanoma-associated retinopathy) [80,83,84].

#### Glaucoma

One of the pathological events in this insidious disease includes declines in RGC function due to IOP elevations compressing nerve fibres traversing through the optic nerve head. Changes in TRP function may contribute to changes in RGC function induced by such stress since they are expressed in this tissue [3,41]. Other tissue changes that may contribute to glaucoma damage are obstruction of aqueous outflow pathways through the trabecular meshwork (TM) and/or a change in ciliary muscle contractility. References describing TRPC channels in these tissues are listed in a recent review [45]. As their activities are sensitive to changes in intracellular Ca^2+^ content, they are referred to as SOCs. It has been suggested that 11 different mechanosensitive TRP channels identified in trabecular meshwork cells could be activated by increases in IOP and contribute to TM pathology [85]. Therefore, developing selective drug targeting mechanosensitive TRP channels may provide a novel option to treat hypertensive glaucoma.

#### Cataract

Increase in intracellular Ca^2+^ level is associated with cataractogenesis [86]. TRPV1 immunostaining was identified in the lens and its activation may contribute to cataractogenesis since such an effect induces rises in intracellular calcium [77]. A possible role is summarized in a review describing store-operated Ca^2+^ channels composed of TRPC subunit expressions (i.e. TRPC1 and TRPC5) in cataract development [47].

#### Ocular tumours

Ocular tumour incidence is rarer than in non-ocular tissues. Choroidal- or corneal tumorigenesis is unique to ocular tissues because it is not directly related to tumour neovascularization, which is a dangerous progression that promotes tumour growth and metastasis. Endothelial cell proliferation drives neovascularization and several different TRP subtypes have been detected in this tissue. However, there are no studies yet describing their contribution to tumour neovascularization.

#### Uveal melanoma

Uveal melanoma (UM) is a devastating disease. Once this tumour metastasizes out of the eye into the liver, lung, bone and skin, patient survival rates are poor. It is the second most prevalent malignant tumour of melanocytes [87,88]. This disease directly develops from degenerated melanocytes in the choroid. TRPM1 was designated as a melanoma metastasis suppressor based on its expression in normal pigment cells in the skin and the eye since it was absent in aggressive, metastasis-competent melanomas [89]. A similar association pertains to TRPA1 because its expression is lower in human UM cells than in healthy uvea [49]. An inverse relationship was described for TRPM8, which is expressed at higher levels in most of the investigated UM cell lines. Furthermore, TRPV1 and the cannabinoid receptor 1 (CB1) are functionally expressed in UM cells. Interestingly, activation of CB1 induced Ca^2+^ transients, which were suppressed by the TRP channel blockers La^3+^ and CPZ whereas capsaicin-induced Ca^2+^ transients could also be suppressed by CB1 activation. Therefore, it is suggested that identification of functional TRPV1, TRPM8, TRPA1 and CB1 expressions in UM may provide novel drug targets for treatment of this aggressive neoplastic disease. A similar suggestion was made for treating non-uveal melanoma (TRPM8) [90].

#### Retinoblastoma

Retinoblastoma (RB) is a malignant retinal tumour, which develops from immature retinal cells. Its incidence is low but is the most common ocular tumour of the eye in children and is associated with a RB mutation [91]. There have been only a few reports describing a TRP’s involvement whereas other types of Ca^2+^ channels have been studied more extensively.

Regarding Ca^2+^ permeable TRPs, Hanano et al. suggested a possible significant regulatory role of TRPM7 for RB cell proliferation as a spontaneously activated Ca^2+^ influx pathway [92]. Another study revealed TRPV1, TRPM8 and TRPA1 gene expression in retinoblastoma cells. Notably, the expression of TRPA1 was suppressed in etoposide-resistant RB cells [46]. Therefore, using genetic approaches to upregulate TRPA1 expression could provide a means to induce etoposide sensitivity and suppress RB cell tumorigenesis.

Furthermore, CB1 receptors could be detected as shown in uveal melanoma cells. Activation of CB1 suppressed the TRPV1-induced Ca^2+^ increases in etoposide-sensitive RB cells whereas this effect did not occur in etoposide-resistant RB cells. Nevertheless, the limited TRP characterizations suggest that there are clear differences between healthy and tumour ocular tissues or cytostatic-resistant ocular tissue. Therefore, modulation of TRP expression and/or function could provide a critically needed therapeutic option. In addition, monitoring TRP expression levels could provide a prognostic marker for identifying this insidious disease.

## Conclusion

Ocular TRP functional expression is essential for mediating both adaptive and maladaptive responses to a wide variety of different environmental conditions that can impact tissue homeostasis maintenance. Table 1 summarizes ocular tissue TRP channel subtype localization and pharmacology described in pertinent references. As there is an association between aberrant TRP channel expression and human disease, these regulators of Ca^2+^ influx may be potential drug targets for usage in a clinical setting. Nevertheless, much effort is still needed to validate this suggestion.

**Table 1:** **TRP channel pharmacology and ocular cell type localization**

**TRP channel subtype**	**Compound**	**Concentration**	**Role**	**Expression/localization**	**References**
TRPC1 -TRPC3	2-APB	100 μM	Antagonist	HCEC Mouse retina TM	[43,61,85]
TRPC4	2-APB	100 μM	Antagonist	RCECMouse retina	[43,61,93]
TRPV1	Capsaicin	1 – 20 μM	Agonist	HCEC^1^, HCK HCEC-12 Rat retina HCjEC Rat and mouse retinas Human retina (tumour) Uvea (tumour)	[3,41,46,49,59,60,66,71,94]
TRPV1	Capsazepine	1 – 10 μM	Antagonist	HCEC HCK HCEC-12 HCjEC	[46,59,60,66,71]
TRPV2	2-APB	200 - 400 μM	Agonist	HCEC-12 HCEC	[71,95]
TRPV2	Camphor	400 μM	Agonist	RPE	[48]
TRPV3	2-APB	200 μM	Agonist	HCEC	[95]
TRPV4	4α-PDD,	5 μM	Agonist	HCEC-12 HCEC^2^, HCjEC	[46,63,70]
TRPV4	GSK 1016790A	5 μM	Agonist	HCEC-12	[70]
TRPM8	Menthol	50 - 500 μM	Agonist	HCNF HCEC-12	[54,55,57,72,96]
TRPM8	Icilin	2 – 60 μM	Agonist	HCK^3,^ HCEC^3^ CEC-12 Human retina (tumour) Uvea (tumour)	[46,49,72,75]
TRPM8	Eucalyptol	3000 μM	Agonist	HCEC-12	[72]
TRPM8	BCTC	10 μM	Antagonist	HCEC-12	[72]
TRPA1	Icilin	2 – 60 μM	Agonist	HCEC-12 TM	[72,75,85]

^1^activation by hypertonic challenge.

^2^activation by hypotonic challenge.

^3^Mergler et al. (unpublished data).

HCEC = human corneal epithelial cells.

RCEC = rabbit corneal epithelial cells.

HCjEC = human conjunctival epithelial cells.

HCK = human corneal keratocytes (stroma).

HCEC-12 = human corneal endothelial cells.

HCNF = human corneal nerve fibres.

RPE = retinal pigment epithelium.

TM = Trabecular meshwork.

Firstly, specific sites on TRP channel structure must be delineated that account for each of the responses mediated by selective channel activity modulation. Secondly, site-specific mutations must be expressed to determine if such alterations correspond with any of the pathophysiological responses by cells that express TRP channels. Finally, drug-induced reversal of any changes caused by disease must be selective without serious side effects. For example, even though TRPV1 inhibition by CPZ can be selective in some settings, this antagonist also elevates body temperature at the same time that it reduces an inflammatory response in some tissues. Accordingly, TRPV1 antagonist development to reduce fibrosis and the likelihood of chronic inflammation may require designing drugs interacting only with specific domains on TRPV1 channels linked to signalling pathways mediating these sight-compromising responses rather than those eliciting body temperature control. As described above, resolving site-specific regions on TRPV1 channels controlling different responses could be beneficial for fine tuning specific responses promoting re-epithelialization rather than those inducing corneal opacification and neovascularization. Judging from the burgeoning number of publications per year, TRP drug targeting may yet improve management in a clinical setting of a of host diseases.

## Abbreviations

TRPA: Transient receptor potential ankyrin
TRPC: Transient receptor potential canonical; TRPM: Transient receptor potential melastatin
TRPML: Transient receptor potential mucolipin
TRPN: Transient receptor potential no mechano-potential
TRPP: Transient receptor potential polycystin
TRPV: Transient receptor potential vanilloid
